# Field Margin Vegetation Structure Buffers Microclimate Extremes in Temperate Agricultural Landscapes

**DOI:** 10.1002/ece3.73899

**Published:** 2026-08-18

**Authors:** Sarah Endicott, Adam Blasl, Marie‐Hélène Brice, Ilona Naujokaitis‐Lewis

**Affiliations:** ^1^ Science and Technology Branch, National Wildlife Research Centre Environment and Climate Change Canada Ottawa Ontario Canada; ^2^ Department of Environmental Management WSP Canada Ottawa Ontario Canada; ^3^ Department of Environmental Science Carleton University Ottawa Ontario Canada; ^4^ Jardin botanique de Montréal Montréal Québec Canada; ^5^ Département de sciences biologiques, Institut de recherche en biologie végétale Université de Montréal Montréal Québec Canada

**Keywords:** agricultural landscapes, climate change, climate change adaptation, ecosystem services, field margins, hedgerow, microclimate, refugia

## Abstract

Field margins in agricultural landscapes range from herbaceous strips to tree‐rich hedgerows, influencing microclimate and wildlife habitat quality. Despite their ecological importance, the microclimatic properties of structurally diverse field margins remain poorly quantified. We investigated how local field margin vegetation structure and landscape context influence microclimatic conditions and assessed how temperature measurement methodology, specifically the use of shielded versus unshielded sensors and the choice of temperature reference, affects estimates of thermal buffering. From June to September 2018, 180 paired shielded and unshielded sensors recorded temperature across 30 fields in 20 one‐kilometre^2^ landscapes located in a temperate Canadian agricultural region, where field margins varied from short herbaceous cover to tall woody canopies. This paired design explicitly quantified how vegetation mediates solar radiation effects and how radiative processes contribute to apparent thermal buffering. We quantified daily maximum and minimum temperature offsets between field margins and adjacent crop fields and compared these measurements to regional weather station data. Linear mixed‐effects models showed that tree‐dominated margins strongly buffered thermal extremes, reducing maximum temperatures and elevating minimum temperatures, while herbaceous and shrubby margins provided limited thermal buffering. Margin width and landscape‐level natural vegetation cover had no detectable effects. Although shielding reduced the magnitude of temperature offsets, the direction of buffering effects remained consistent. Temperature differences between margins and fields were substantially larger when compared to local in‐field sensors rather than regional weather stations. Our findings demonstrate that both vegetation structure and measurement methodology substantially influence estimates of microclimate conditions, with implications for quantifying thermal buffering in agricultural landscapes.

## Graphical abstract

Tree‐rich field margins strongly buffered thermal extremes, lowering maximum and raising minimum temperatures compared to adjacent crop fields. Paired shielded and unshielded sensors showed how vegetation modifies thermal conditions and how measurement approach influences estimates of thermal buffering, supporting the potential role of treed margins as potential microrefugia.

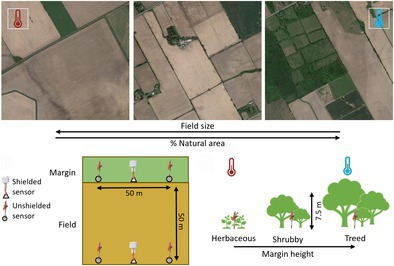

## 1 Introduction

In an era of rapid climate change, identifying areas and landscape features that remain relatively buffered from temperature extremes may reduce risks associated with climate warming (Lenoir et al. 2017; Riddell et al. 2021; Suggitt et al. 2015). Forests, in particular, buffer temperatures, resulting in understory microclimates that are different from free‐air macroclimates as measured by weather station data (Chen et al. 1999; De Frenne et al. 2021, 2019; Scheffers et al. 2014). Forest microclimates support microrefugia, areas which enable species to persist locally due to suitable microclimate conditions that remain buffered from climate change impacts (Ashcroft 2010; Dobrowski 2011; Kemppinen et al. 2024). The microrefugia potential of larger tracts of contiguous forests and forest‐dominant ecosystems is well documented (De Frenne et al. 2021; Greiser et al. 2018; Haesen et al. 2023; Morelli et al. 2020; Wei et al. 2024; Zellweger et al. 2020). However, with much of the global land mass consisting of agriculture (Foley et al. 2005; Ramankutty et al. 2008), understanding how individual trees, small forest remnants, hedgerows, and other non‐crop vegetation create buffered microclimates and potentially function as microrefugia remains a key priority for strengthening climate resilience in agricultural systems.

Agricultural landscapes can contain a wide range of land uses and land cover types, which can affect the availability of microclimates. There is substantial regional variation across agricultural systems, with these human‐dominated landscapes varying from intensive agricultural systems with large fields of monoculture crops and few non‐crop cover types to landscapes consisting of a diversity of crops and non‐crop ‘natural’ covers, such as remnant forest patches, wetlands and field margins (Foley et al. 2005; Tilman et al. 2001; Tscharntke et al. 2012). Field margins, defined as relatively narrow linear strips of natural and semi‐natural vegetation that separate crop or pasture fields, are common features of many agricultural landscapes that contribute to a diversity of ecosystem services (Burel 1996; Haddaway et al. 2018; Montgomery et al. 2020; Priyadarshana et al. 2021). Field margins, and treed hedgerows specifically, function as windbreaks and reduce soil erosion, resulting in improved crop yields (Forman and Baudry 1984; Haddaway et al. 2018). Other functions include pest regulation and pollination services (Montgomery et al. 2020; Poveda et al. 2012; Précigout and Robert 2022), ecological corridors (Forman and Baudry 1984) and habitat for biodiversity (Montgomery et al. 2020; Poveda et al. 2012; Précigout and Robert 2022; Priyadarshana et al. 2021). With accelerating climate change, benefits of non‐crop natural vegetation in agricultural landscapes extends to their role in climate change mitigation as demonstrated by their carbon sequestration potential (Drexler et al. 2024). From a climate change adaptation perspective, field margins can introduce microclimate heterogeneity into agricultural landscapes, thereby providing thermally buffered habitats that may function as microrefugia for biodiversity (Keppel et al. 2015) and contribute to local climate regulation (Burkhard et al. 2012).

While the role of larger contiguous forests in creating unique microclimates has gained traction recently (De Frenne et al. 2021; Haesen et al. 2023; Jucker et al. 2020), the contribution of field margin vegetation to microclimate heterogeneity in agricultural landscapes remains less well studied (Haddaway et al. 2018). Local scale attributes of field margins, such as vegetation structure and composition, can affect the microclimates they create (Ghafarian et al. 2024; Sánchez et al. 2009; Vanneste et al. 2020). In a continent‐wide study in Europe, Vanneste et al. (2020) found that hedgerows with taller, wider and denser tree cover produced cooler microclimates that buffered temperature extremes, although buffering within hedgerows was lower than in adjacent forest woodlots. The few studies assessing field margin microclimates in an agricultural context have predominantly focused on woody vegetation (Ghafarian et al. 2024; Sánchez et al. 2009; Vanneste et al. 2020); yet field margin vegetation can vary from herbaceous plants to shrubs to trees, depending on factors including land conversion history and agricultural management practices. To our knowledge, studies of microclimates in field margins have taken place in Europe, which has a long history of the use of hedgerows (Collier 2021; Forman and Baudry 1984). Other agricultural landscapes, however, have different land use histories and distinct past and future climate contexts. Thus, gaps remain in our understanding of the effects of the full range of field margin vegetation on microclimates and the applicability of previous studies to other regions.

The spatial heterogeneity of agricultural landscapes necessitates considering landscape scale factors, in addition to local scale attributes, when examining microclimates. The microclimate influence of field margin vegetation can extend beyond the spatial boundaries of the margin itself. For example, small woody vegetation associated with field margins can cool land surface temperatures in adjacent crop fields (Ghafarian et al. 2024). At the regional scale, land use and land cover can affect mesoclimates and local temperatures. The impact of agriculture and forest land covers on surrounding landscape climate is a complex combination of cooling through evapotranspiration, warming due to lower albedo, and changes in wind speed (Mahmood et al. 2014). These processes are influenced by vegetation type and structure, as well as moisture availability. Several recent studies have found that landscape scale forest cover has a cooling effect (Borderieux et al. 2023; Peng et al. 2020; Zimmermann et al. 2024). For example, Peng et al. (2020) found that the amount and configuration of different land cover types affected temperature, with water, forestland and shrubland contributing to cooling. These landscape scale effects may modify the influence of field margin vegetation on temperature but have not been included in previous studies on this topic.

In addition to issues of spatial scale, recent research on microclimate measurement has generated debate about how air temperatures should be measured and compared across locations (Ashcroft 2018; Maclean et al. 2021; Terando et al. 2018, 2017). When determining whether a landscape feature creates a distinct microclimate, conditions within that feature are typically compared with a macroclimate reference, commonly provided by a nearby weather station (De Frenne et al. 2021; Zellweger et al. 2024). Weather stations measure air temperature 2 m above the ground, are shielded against solar radiation and are positioned to minimise impacts of local terrain or vegetation (Bramer et al. 2018; Maclean et al. 2021). However, these coarse scale weather station measurements do not reflect the range of microclimatic conditions that organisms experience in near‐surface, vegetation‐mediated habitats (Bramer et al. 2018; Pincebourde et al. 2007; Potter et al. 2013).

Interest in microclimates has increased the use of compact temperature sensors (e.g., HOBO loggers, iButtons, etc.) in place of weather station measurements to capture temperatures at the spatial scale and within the microhabitat experienced by organisms (De Frenne et al. 2025). These sensors have been used in a wide range of applications including measurements of soil, air, surface and water temperatures (see Table S4 in Terando et al. 2017), and are frequently used to compare microclimate air temperature with those measured by weather stations (Gilbert et al. 2022; Latimer and Zuckerberg 2017; Vanneste et al. 2020). However, Maclean et al. (2021) and Terando et al. (2017) raised concerns that unshielded or poorly shielded compact sensors are exposed to solar radiation and temperatures may be biased when compared to weather station measurements. From an ecological perspective, however, solar radiation can be considered an integral component of the thermal environment experienced by organisms, while access to shade can reduce this exposure and facilitate behavioural thermoregulation (Elmore et al. 2017; Kearney et al. 2009). In response to Terando et al. (2017), Ashcroft (2018) argues that, because organisms are exposed to solar radiation, reducing its influence through sensor shielding could instead bias measurements towards cooler temperatures. These contrasting perspectives emphasise the importance of understanding how methodological choices related to air temperature measurements influence the interpretation of results.

Here, we used fine scale temperature measurements to investigate how local vegetation structure, landscape context and radiative exposure influence temperature offsets produced by field margin vegetation across temperate agricultural landscapes in Ontario, Canada. Our primary objective was to quantify the magnitude of thermal buffering provided by field margins relative to conditions in adjacent agricultural crop fields. We measured daily and hourly temperatures at paired field margin and crop field locations using both shielded and unshielded microclimate sensors, allowing us to calculate temperature offsets and to partition air temperature modification from radiative (solar) heating effects. Because offsets are commonly calculated relative to macroclimate conditions, we compared our microclimate measurements with regional weather station data and evaluated how the choice of temperature reference influences estimates of thermal buffering. We assessed how temperature offsets varied with local vegetation structure (field margin height and width) and landscape scale natural vegetation cover. By directly contrasting shielded and unshielded sensors, we tested how vegetation cover mediates radiative heating and evaluated how this methodological choice influences estimates of thermal buffering. We expected that taller, wider field margins would exhibit greater cooling relative to adjacent fields and weather stations in open areas, that greater landscape scale natural vegetation cover would enhance cooling, and that differences between shielded and unshielded sensors would be greatest in open, unshaded environments.

## 2 Methods

### 2.1 Study Area and Site Selection

**FIGURE 1:**
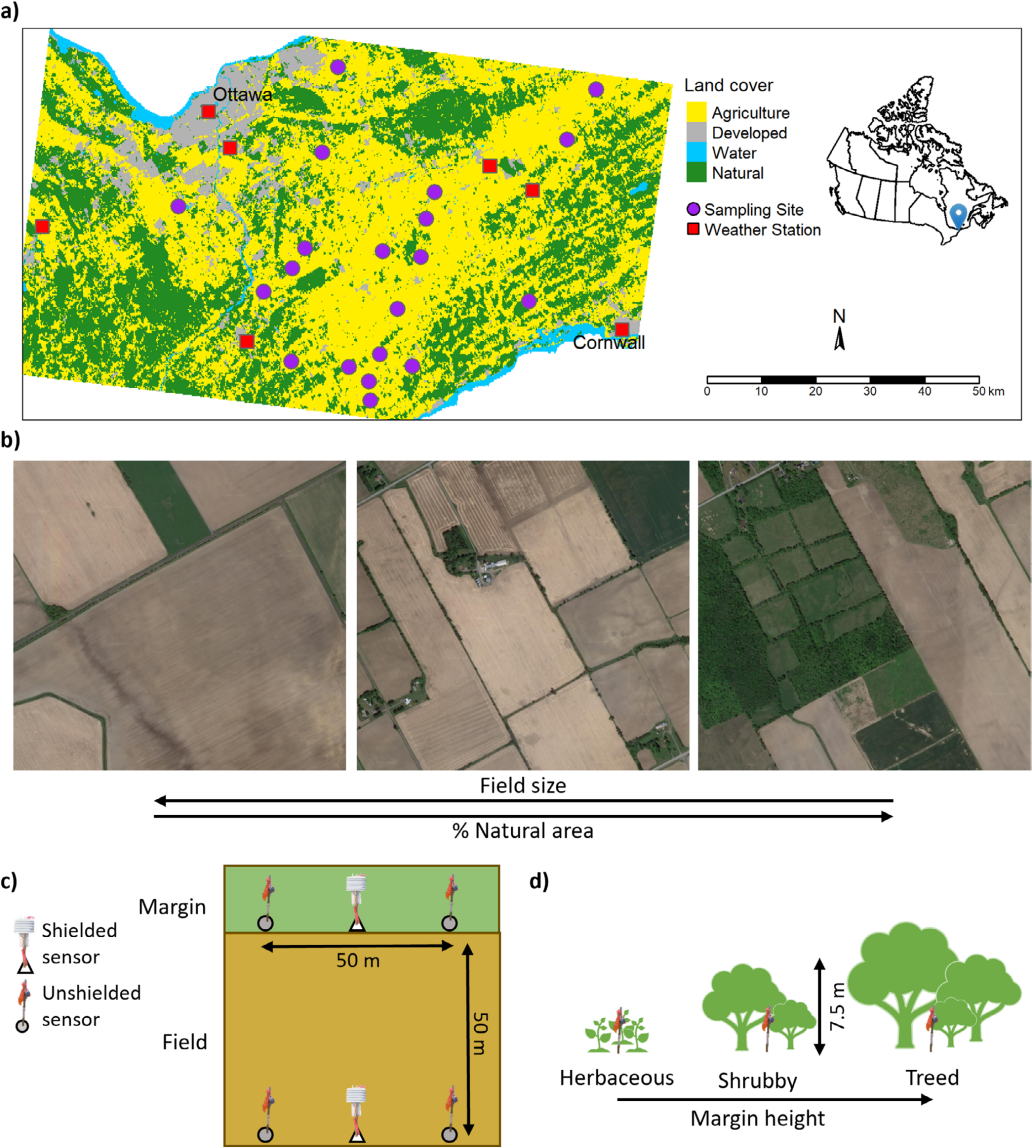
(a) Map of the study area in eastern Ontario, Canada including the locations of the 7 weather stations and 20 sampling sites. Landcover is based on the Southern Ontario Land Resource Information System Version 3.0. *Source:* Ontario Ministry of Natural Resources and Forestry King's Printer for Ontario, 2023. (b) Examples of 1 by 1 km landscapes that sampling sites were located within, showing decreasing mean field size from left to right at the landscape scale and increasing proportions of natural area from left to right. Satellite image source: Imagery 2022 Google, Imagery 2022 CNES/Airbus, Maxar Technologies (c) A diagram showing the placement of sensors along the margin and in the field. (d) A diagram showing the sensor placement within the margin and the range of margin vegetation heights that were included.

Our study was located across a 10,000 km^2^ area of southeastern Ontario, Canada, 114 m above sea level (45°19′ N, 75°40′ W) (Figure 1a). The region is largely agricultural with corn and soybean crops dominating the landscape. These two crops are typically rotated in alternate years, with wheat, barley and forage crops, such as alfalfa, clover and hay, occasionally included in rotations (Statistics Canada 2017). Although much of the region has been converted to agricultural, urban and suburban land uses, the remaining forest cover consists of mixed deciduous and coniferous forests. The mean annual temperature in this region is 6.4°C, and mean annual precipitation is 943.4 mm. During the summer months (June through to the end of September) the mean temperature is 18.5°C and mean total precipitation is 360.3 mm (https://climateatlas.ca/map/canada).

We selected 20 1 km^2^ landscapes that maximised variation in mean crop field size and captured variation in the amount of natural land covers surrounding fields and across the landscape (Figure 1b). Site selection included a subset of landscapes used in Fahrig et al. (2015) to take advantage of ongoing parallel studies on pollinators. Each landscape was separated by a minimum distance of 3.5 km. Within the 20 sites, we selected one to two fields, resulting in a total of 30 fields to assess microclimate heterogeneity. Temperature sensors were deployed along transects spanning a focal crop field (hereafter, field) and an adjacent field margin.

At the field scale, we applied multiple criteria to select paired field‐field margin sampling locations. First, we selected fields that were bordered by a minimum of three vegetated field margins, with adjacent land‐use consisting of another crop field, and without drainage ditches or waterways within the field margin. Second, across all sites, we selected field margins to represent a gradient of vegetation composition and heights, ranging from herbaceous to woody vegetation, including shrubs and trees. Where possible we selected fields where all surrounding field margins were of similar height and vegetation type. Third, we prioritised soybean fields due to their lower crop height which facilitated field temperature sensors deployments. However, when this was not possible other low height crops (e.g., hay, wheat, barley, etc.) were chosen. Corn fields were excluded due to their potential height, which could overgrow the sensors and alter field temperature measurements.

### 2.2 Microclimate and Macroclimate Data

Within each focal field, we created two 50 m transects, one in the margin and another 50 m into the crop field. Along each transect we deployed three sensors every 25 m (Figure 1c). When the field size was smaller than 100 m in length, the transect was placed in the centre of the field to avoid being too close to the opposite hedgerow. A total of 180 temperature sensors were installed within 30 paired transects, and were deployed during the 2018 growing season, between June 11th and June 26th, and removed between September 14th and September 21st. We used HOBO temperature loggers (112 loggers of model MX2201 and 68 loggers of model UA‐002‐08; range −20°C to 70°C; accuracy ±0.5°C and ±0.53°C, respectively). All temperature sensors were assessed for accuracy using a water bath and an independent thermometer in the lab prior to deployment. Each temperature sensor was mounted 1 m above the soil surface on a wooden stake and was configured to record at 30 min intervals. Temperature sensors in the field were oriented towards the margin and margin sensors were oriented towards the field to minimise variation, and field margins were selected to minimise variation in general orientation (i.e., transects were selected to run in a north–south direction, as best as possible). To test the effect of shielding sensors, along each transect two temperature sensors were unshielded and one was shielded using Onset RS1 Solar Radiation Shields, resulting in 60 shielded and 120 unshielded sensors (Figure 1c).

We obtained macroclimate temperature data for each study site using nearby weather stations from Environment and Climate Change Canada. We selected all weather stations located within a buffer of 30 km around the entire study area resulting in seven weather stations (Figure 1a). Using the R package “weathercan” (LaZerte and Albers 2018), we retrieved hourly temperature data from the four stations for which these data were available and calculated daily mean, minimum and maximum temperatures from all seven stations over the study period. We calculated the macroclimate temperature as the inverse distance weighted mean across the weather stations for each temperature measure at each study site.

### 2.3 Environmental Variables

We measured field margin height using a clinometer at each of the three sensor locations in the field and calculated the mean. Margin width was measured at each of the three field margin sensor locations, and the mean was calculated. Margin width extended from the edge of the sampled focal field, across the field margin, to the edge of the crop in the adjacent field. The proportion of natural vegetation within each 1 km^2^ site was based on field surveys of each focal landscape conducted in 2018. We used aerial imagery from 2011 and 2012 with a spatial resolution of 40 cm (see Fahrig et al. 2015), which we manually updated using field observations. The natural land cover layer consisted of non‐cropped areas, including field margins, forests, wetlands and open water.

### 2.4 Data Analyses

#### 2.4.1 Temperature Offsets by Sensor Location

We evaluated the differences between microclimates and macroclimate by comparing temperatures recorded by sensors located in fields (F) and margins (FM) to each other and to the macroclimate (M) temperature derived from weather station data. Temperature differences were quantified by calculating three temperature offsets:

$$T_{\text{offset}\;\mathrm{FM}-F}=T_{\mathrm{FM}}-T_{F}$$

$$T_{\text{offset}\;\mathrm{FM}-M}=T_{\mathrm{FM}}-T_{M}$$

$$T_{\text{offset}\;F-M}=T_{F}-T_{M}$$

The temperature offset is the difference between temperatures measured by different sensors, while buffering refers to when conditions in a particular microclimate are less extreme than temperatures in a different microclimate or than the macroclimate.

Of the 180 temperature sensors, seven failed and were removed. In addition, 19 time series were truncated thus the total number of temperature sensors varied between 155 and 173 over the course of the season. Microclimate temperature records were truncated to the period from June 27th to September 13th, 2018 to capture the dates when all sensors were deployed. Temperature records were averaged across the two co‐located unshielded sensors. We summarised the 30 min temperature readings for each sensor into daily minimum, mean and maximum temperature. We also calculated daily average absolute deviation (AAD), a measure of daily temperature variation, as the mean of the differences between each data point and the median.

To test for differences between temperatures recorded at different locations we performed paired Wilcoxon signed rank tests to determine if the difference between two sensors was symmetrical around zero and to estimate the median offset and 95% confidence intervals. For each daily temperature measure (minimum, mean and maximum) we grouped the data by the location of the sensor (field margin, field, or macroclimate) and shield status of the sensor. For field margin sensors, we compared data grouped by shield status and field margin height. Field margin height was classified as short (< 7.5 m) or tall (≥ 7.5 m), based on minimum height estimates for shrubs and small trees (Petrides 1972). This value also coincided with natural breaks in our vegetation height data (Figure S1).

#### 2.4.2 Effect of Local and Landscape Characteristics on Offset

We assessed how the temperature offset between field margins and fields changed as a function of local and landscape factors. For each paired transect, $T_{\text{offset}\;\mathrm{FM}-F}$ was calculated for each of the three temperature components: daily mean, minimum and maximum temperatures. Negative offset values reflect cooler temperatures in field margins compared to fields while positive values indicate warmer temperatures in field margins. As such we interpret negative offset values for maximum temperature and positive offset values for minimum temperature as indicative that field margins are providing buffering against extreme temperature values in the surrounding landscape.

We fit linear mixed‐effect models (LMMs) for each daily temperature metric with $T_{\text{offset}\;\mathrm{FM}-F}$ as the response variable and field margin height, field margin width, percent natural vegetation in the landscape and macroclimate temperature as fixed effects (Table 1). We also included interactions between margin height and width, margin height and percent natural vegetation and margin width and percent natural vegetation. We included site ID as a random effect to account for non‐independence between repeated samples from the same site. Separate models were fitted for unshielded and shielded sensors. Based on exploratory data analysis, field margin height showed a stepped relationship to temperature offset. To explore this, we fit two versions of the model, one with a quadratic term for margin height, and another where margin height was specified as a binary variable (< 7.5 m vs. ≥ 7.5 m). We compared the two versions of the model based on the Akaike's Information Criterion (AIC).

**TABLE 1:** Description and spatial scale of variables used in the linear mixed‐effect models.

Variable	Description	Spatial scale
Field margin height	Mean field margin height, measured using a clinometer from each of the three field sensor locations (m).	Local
Field margin width	Mean field margin width, measured across the margin at the three sensor locations (m).	Local
Percent natural vegetation cover	Proportion of natural vegetation within the 1 km^2^ landscape surrounding the focal field (%).	Landscape
Macroclimate temperature	Inverse distance weighted mean of the temperature from weather stations within 30 km of the study area (°C).	Regional

To account for temporal auto‐correlation in our data we added correlation structures to our models. We ran each model with (1) no correlation structure, (2) an auto‐regressive model of order 1 (corAR1) auto‐correlation structure and (3) an autocorrelation‐moving average correlation structure of order *p* = *q* = 1 (corARMA). We compared models using AIC (Zuur et al. 2009). We incorporated corARMA as a model parameter based on evidence that including corARMA auto‐correlation structure improved model fits and reduced temporal auto‐correlation (Figure S4).

We evaluated model performance using marginal *R* ^2^ for linear mixed effect models (Nakagawa and Schielzeth 2013) and diagnostic plots. We assessed multicollinearity among model predictors by calculating their variance inflation factors using the package “car” (Fox and Weisberg 2019) and confirming that all values were less than 2. The R package “nlme” was used to fit our LMMs (Pinheiro et al. 2023). All statistical analyses and visualisations were performed using the open source statistical software R version 4.4.1 (R Core Team 2024).

## 3 Results

**FIGURE 2:**
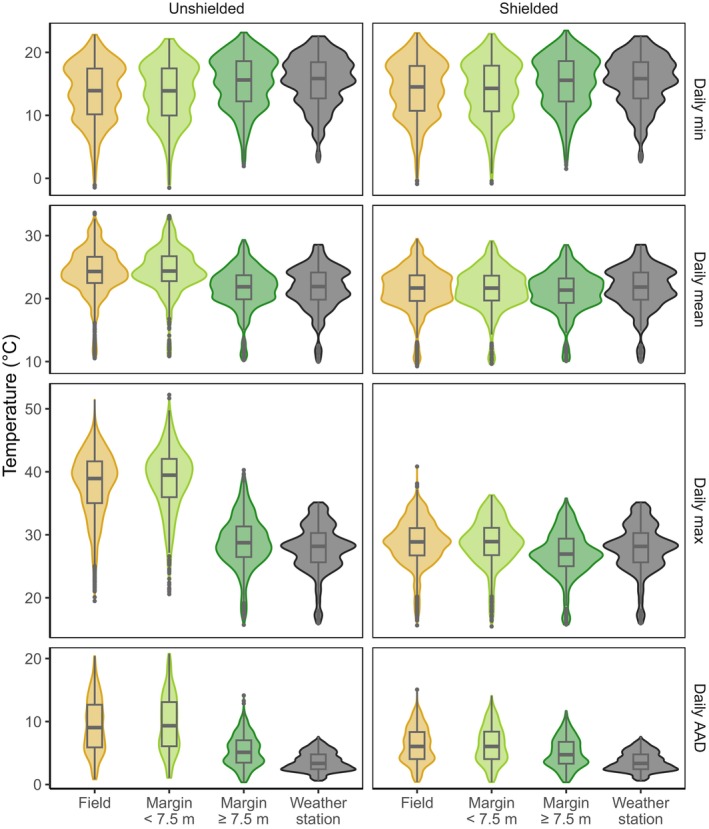
Comparison of the distributions of mean daily temperature metrics among fields, field margins classified as tall (≥ 7.5 m) or short (< 7.5 m) vegetation height, and macroclimate measurements derived from weather station data, shown for unshielded (left) and shielded sensors (right). AAD denotes average absolute deviation. The scale is consistent across panels, although different portions of the scale are shown in each panel.

On average, sensors in field margins recorded less extreme temperatures than sensors in fields across the surveyed agricultural landscapes as indicated by lower daily mean and maximum temperatures, higher daily minimum temperatures and within day temperature variability (i.e., lower diurnal range and average absolute deviation) (Figure 2; Table S1). For example, the mean daily maximum temperature recorded by unshielded sensors was 38.03°C in fields, compared to 32.59°C in margins. For shielded sensors, corresponding values were 28.73°C in fields and 27.67°C in margins. Macroclimate temperatures derived from weather station data were more similar to those recorded by shielded sensors in field margins and less extreme than those recorded by sensors in fields, with a mean daily maximum temperature of 27.83°C.

**FIGURE 3:**
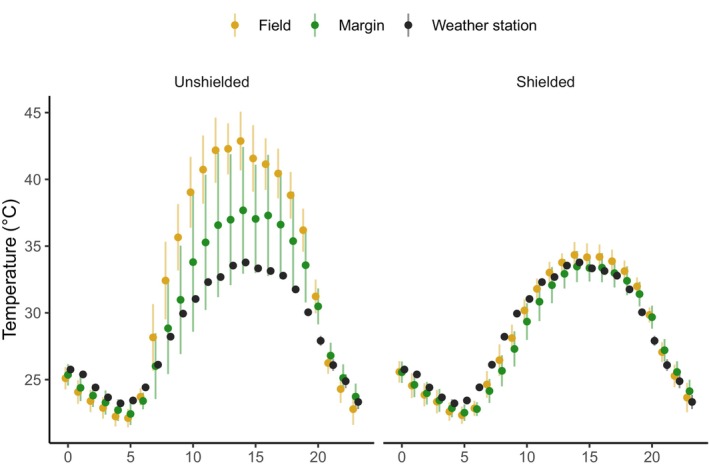
Mean hourly temperature across all sites on July 1st 2018 for sensors in fields, margins and weather stations. Lines show plus and minus one standard deviation and indicate the variation among sites. Note that only four of the seven weather stations used in our study had hourly data available.

Hourly temperature patterns revealed that temperature offsets varied throughout the day (Figure 3). Macroclimate temperatures were warmer than those recorded by other sensors in the early morning until about noon for shielded sensors, after which point field and margin temperatures became slightly warmer. In contrast, temperatures measured by unshielded sensors in both fields and margins increased much faster, exceeding macroclimate temperatures by 7 h to 20 h. Sensors in fields had the largest offset from the macroclimate while margins showed smaller offsets on average, but much more variation among sites. Overall, macroclimate temperatures were the most stable throughout the day, which is also reflected by the lower average absolute deviation (Figure 2; Table S1).

**FIGURE 4:**
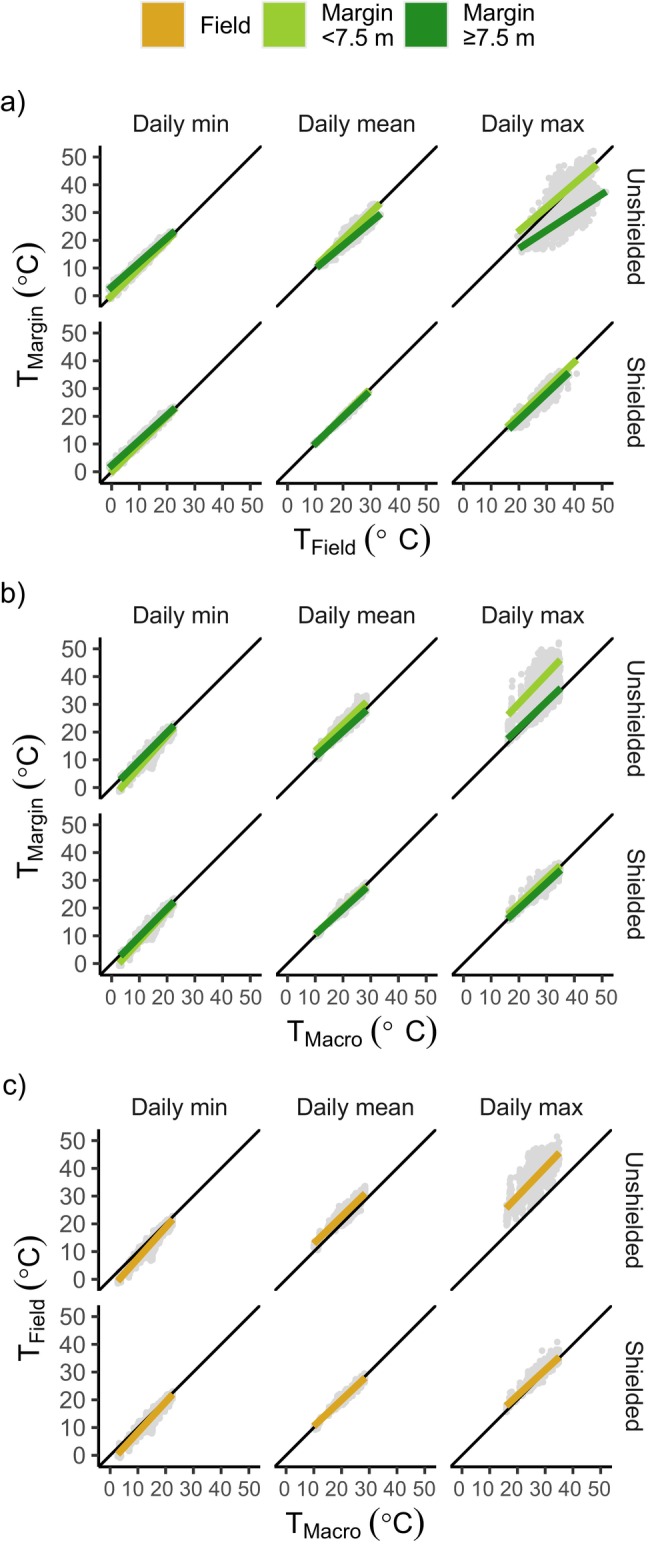
Comparison of mean daily temperature metrics among (a) margins and fields, (b) margins and macroclimate, and (c) fields and macroclimate. In panels (a) and (b), light green denotes margins with vegetation < 7.5 m tall, and the dark green denotes margins with vegetation ≥ 7.5 m tall. Coloured lines show univariate linear relationships between paired temperature measurements. The 1:1 diagonal line indicates no temperature offset; points above the diagonal indicate a positive offset while points below have a negative offset.

Field margins provided significant buffering of extreme temperatures observed in fields, with tall hedgerows (≥ 7.5 m) providing the strongest buffering (Figure 4a; Figure S2; Table S2). Daily maximum temperatures were significantly cooler in margins than in fields for both shielded (median offset = −0.99, CI = −1.05 to −0.92) and unshielded sensors (median offset = −5.49, CI = −5.74 to −5.25). This cooling effect was only present for tall field margins (Shielded: median offset = −1.65, CI = −1.72 to −1.59; Unshielded: median offset = −9.26, CI = −9.42 to −9.10) while temperatures in short field margins were slightly warmer or the same as in fields (Shielded: median offset = 0, CI = −0.04 to 0.04, Unshielded: median offset = 0.52, CI = 0.33 to 0.72).

A similar but weaker pattern was observed for daily minimum temperatures. Overall, field margins were significantly warmer than fields for both shielded (median offset = 0.49, CI = 0.45 to 0.54) and unshielded (median offset = 0.96, CI = 0.91 to 1.01) sensors. When hedgerow height was considered, only tall hedgerows were warmer (Shielded: median offset = 0.90, CI = 0.86 to 0.94; Unshielded: median offset = 1.58, CI = 1.53 to 1.63) and short hedgerows were the same or cooler (Shielded: median offset = −0.043, CI = −0.09 to −0.02; Unshielded: median offset = −0.02, CI = −0.05 to 0.00).

Macroclimate temperatures tended to be less extreme than temperatures recorded in fields and field margins (Figure 4b,c; Figure S3; Table S2). For daily maximum temperature, both fields and field margins were warmer than the macroclimate for unshielded sensors (Field margin: median offset = 4.64, CI = 4.37 to 4.89; Field: median offset = 10.21, CI = 10.08 to 10.337), while for shielded sensors, fields were warmer (median offset = 0.79, CI = 0.74 to 0.84) and field margins were cooler (median offset = −0.24, CI = −0.30 to −0.17). When field margins were separated by height, tall field margins were only slightly warmer than the macroclimate for unshielded sensors (median offset = 0.88, CI = 0.78 to 0.98) and cooler for shielded sensors (median offset = −0.89, CI = −0.94 to −0.83). On the other hand, short field margins were similar to fields, with unshielded sensors being much warmer (median offset = 10.70, CI = 10.48 to 10.91) and shielded sensors only slightly warmer (median offset = 0.80, CI = 0.72 to 0.87) than the macroclimate. Unlike when compared to fields, there was no buffering of minimum temperature in field margins compared to macroclimate temperatures (median offset CIs between −0.08°C and −1.91°C depending on shielding and field margin height).

**FIGURE 5:**
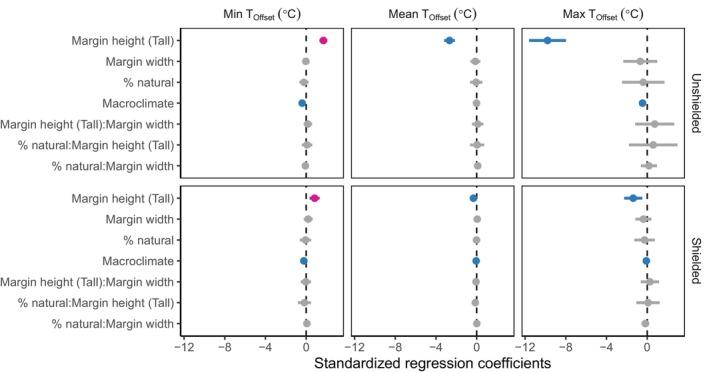
Standardised regression coefficients and 95% confidence intervals from linear mixed‐effect models estimating the effect of local and landscape variables on temperature offset metrics (minimum, mean, maximum) for both unshielded and shielded sensors. Temperature offsets represent the difference between field margins and fields ($T_{\text{field margin}}-T_{\text{field}}$). Positive regression coefficients (red) indicate covariates associated with warmer temperatures in field margins, negative coefficients (blue) indicate covariates associated with cooler temperatures, and estimates in grey have confidence intervals that include zero.

Temperature offsets ($T_{\text{offset}\;\mathrm{FM}-F}$) for daily minimum, mean and maximum temperature for both shielded and unshielded sensors were significantly influenced by field margin height, with positive effects for minimum temperature and negative effects for mean and maximum temperature, suggesting that tall trees buffer extreme temperatures (Figure 5; Tables S3 and S4). Field margin width, landscape scale natural cover and all interactions (% natural vegetation:field margin width, % natural vegetation:tall field margin, tall field margin:field margin width) had negligible effects across metrics and shielding status (Figure 5; Tables S3 and S4). Across all three temperature metrics and regardless of shield status, $T_{\text{offset}\;\mathrm{FM}-F}$ was significantly negatively related to free‐air macroclimate temperature, except for mean temperature of unshielded sensors where the effect was minimal (Figure 5; Tables S3 and S4).

**FIGURE 6:**
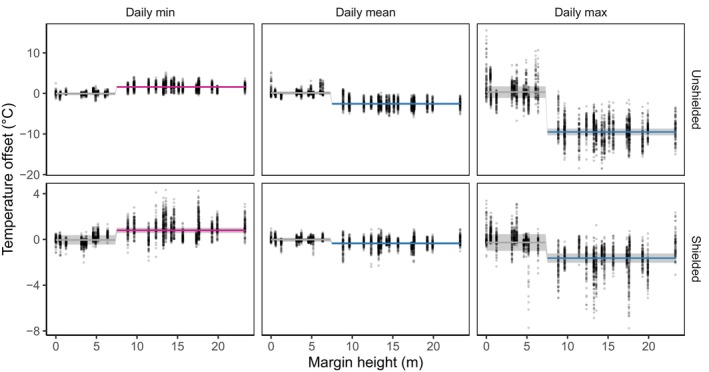
Conditional effect of field margin height on $T_{\text{offset}\;\mathrm{FM}-F}$ with 95% confidence intervals for unshielded and shielded sensors based on the results of linear mixed‐effect models. The effect shown is conditional on other variables in the model being held at their means. Points show the raw data. Red and blue lines indicate warmer and cooler temperatures in hedgerows, respectively, while grey lines indicate relationships where the confidence interval crosses zero.

We fitted models using both continuous and binary versions of field margin height. Field margin height was significant in both models but we present results for the binary version here, as it provides a closer visual match to the raw data (Figure 6). For model results with continuous height as a covariate see Supporting Information (Tables S5 and S6). The binary field margin height variable reflected that $T_{\text{offset}\;\mathrm{FM}-F}$ appeared to have a threshold between 6.3 and 8.9 m and was close to 0 for field margin heights < 7.5 m and was further from 0 for heights ≥ 7.5 m (Figure 6).

## 4 Discussion

Using a dataset of paired measurements of summer temperatures in crop fields and adjacent field margins across agricultural landscapes in Ontario, Canada, we found that treed margins create thermally buffered microhabitats in temperate agricultural landscapes, primarily through local vegetation structure rather than landscape scale tree cover. Across the sampling period, the mean difference in daily maximum temperature between field margins and fields, located only 50 m apart, was 1.06°C for the shielded sensors and 5.44°C for unshielded sensors. Both the mean and maximum temperature were cooler in hedgerows compared to agricultural fields, while minimum temperatures were higher and average absolute deviation was lower. Temperature offsets also varied throughout the day, with the largest differences occurring in the afternoon. Together these patterns indicate that field margins, particularly those consisting of shrubs and trees, thermally buffer the environment relative to agricultural fields. Importantly, our results further demonstrate that methodological decisions in temperature measurement, such as whether sensors are shielded, can substantially influence both the magnitude of observed effects and the conclusions drawn. These findings underscore the importance of carefully considering measurement approaches when interpreting hedgerow cooling effects, which we explore in detail below.

Our study builds on existing microclimate research by explicitly disentangling the relative influence of local field margin vegetation height versus landscape scale natural vegetation cover on the availability of microclimates and potential thermal refugia. To date, empirical studies of field margin microclimates in agricultural landscapes have been conducted predominantly in European systems (e.g., Vanneste et al. 2020); by focusing on North American agricultural landscapes in a temperate region, we extend this work to a region with distinct land use histories, field configurations and management contexts. In addition, our in‐field microclimate logger deployments extend recent advances in microclimate measurement and mapping human‐modified agricultural landscapes rather than focusing primarily on forested or semi‐natural habitats (Zellweger et al. 2024).

Overall, our results aligned with previous studies of forest and field margin (i.e., hedgerow) microclimates and demonstrate the critical role of vegetation structure for thermal buffering. Our median maximum temperature offset for tall field margins, using weather stations (i.e., macroclimate) as the reference (−0.89°C), was comparable with Vanneste et al. (2020) (−0.72°C) and Zellweger et al. (2024) (−0.7°C) for field margins and, as expected, smaller than their measured differences in forests (−1.52°C and −1.3°C, respectively). However, using in‐field sensors as the reference revealed substantially larger buffering effects (−1.66°C for shielded sensors), better capturing the thermal contrast organisms experience between hedgerows and adjacent agricultural fields. On summer days trees intercept solar radiation and contribute to cooling by evapotranspiration, while also blocking wind and preventing mixing with warmer air (Geiger et al. 1995). At night trees can act as insulation by intercepting heat released from the ground (Geiger et al. 1995). These processes are consistent with our finding that field margin height strongly influences thermal buffering, echoing studies showing that taller vegetation provides greater cooling capacity (Carroll et al. 2016; Vanneste et al. 2020). While Vanneste et al. (2020) found field margin width contributed to buffering capacity, in our models neither field margin width nor the interaction of width and height was significant. This lack of effect likely reflects the limited variation in widths, where 63% of field margins were between 5 and 15 m wide and likely subject to edge effects (Corbit et al. 1999).

While our study demonstrated substantial thermal buffering within treed field margins, we found no detectable effect of landscape scale natural cover (measured within 1 km^2^ surrounding loggers). This suggests that the cooling benefits of trees in the temperate agricultural landscapes operate primarily at local scales rather than through landscape scale temperature moderation, at least within the range of tree cover and spatial configurations present in our temperate study system. Several mechanisms may explain this pattern. First, evapotranspiration driven cooling effects from trees are highly localised, diminishing rapidly from tree canopies (Ghafarian et al. 2024; Ziter et al. 2019; Schmidt et al. 2019). Our findings align with these studies showing that small woody features have highly localised cooling effects. Second, fragmented forest cover and intervening open areas in agricultural landscapes may limit the development of landscape or regional cooling, as the mosaic structure of small fields, field margins and small remnant forest patches create complex spatial variation in cooling, patterns (Mendes and Prevedello 2020; Schmidt et al. 2019). Third, although temperate forests generally provide net cooling through evapotranspiration that outweighs albedo effects, the magnitude of effects is context dependent (Anderson et al. 2011; Lee et al. 2011; Li et al. 2015). Detectable landscape scale cooling may require sufficient tree cover, patch size, or spatial continuity; evidence from urban, rural and forest systems suggests these attributes shape cooling effects (Anderegg et al. 2020; Bright et al. 2017; Ziter et al. 2019), but the extent to which these relationships operate in temperate agricultural landscapes remains poorly studied. Finally, the relatively low overall natural cover in our landscapes (< 40%) may have limited our ability to detect landscape‐scale effects. Further research is needed to better understand whether thresholds in forest cover proportion and spatial configuration influence landscape‐scale temperature patterns in agricultural regions.

The ecological interpretation of microclimates depends on how temperature is measured across contrasting field margin vegetation structure and landscape contexts. Solar radiation and wind speed play important roles in the way organisms experience their microclimate (Helmuth et al. 2010). Weather stations, by contrast, are designed to limit the effect of these drivers and measure macroclimate air temperature under standardised conditions (Lembrechts et al. 2019; Potter et al. 2013). This creates a fundamental disconnect when applying weather station data to ecological questions. Some organisms in agricultural landscapes experience solar radiation as an integral component of their thermal environment, whereas weather stations are explicitly designed to exclude these effects. Recent studies have raised concerns that temperature sensors and shielding techniques commonly used in ecology can be biased by solar radiation when deployed in open exposed areas (Gril et al. 2023; Maclean et al. 2021; Terando et al. 2017). However, including radiative heating effects in microclimate measurements may be appropriate when the goal is to understand how organisms experience thermal variation across habitats. To explore this in our system, we explicitly compared shielded and unshielded sensors deployed at the same locations across habitat types, allowing us to quantify the magnitude and context‐dependency of radiative heating effects.

Our shielding experiment revealed that radiative heating bias is strongly mediated by vegetation structure within agricultural landscapes. Differences between shielded and unshielded sensors were substantial in crop fields (approximately 9°C for daily maximum temperatures) but much smaller in treed field margins and during nighttime conditions, as reflected in daily minimum temperatures. This suggests that radiative heating bias is minimal in shaded environments and absent during nighttime conditions. These patterns are consistent with Terando et al. (2017) and Maclean et al. (2021), who observed similar results with compact sensors in open habitats. When compared to the nearby weather station, shielded and unshielded sensors in crop fields recorded warmer daily maximum temperatures (0.8°C and 10°C warmer, respectively), likely reflecting both residual sensor radiative heating and genuine microclimatic differences between crop canopies and the standardised weather station placement.

We assessed two complementary aspects of thermal conditions associated with hedgerows: temperatures recorded by shielded sensors, which are less affected by radiative heating, and temperatures recorded by unshielded sensors, which reflected the combined effects of air temperature and radiative exposure. This perspective guided our comparative study, in which we quantified relative temperature differences between field margin vegetation height and adjacent crop fields using paired sensors. The choice of temperature reference substantially affected the apparent magnitude of vegetation‐mediated thermal buffering. Temperature differences between field margins and reference environments were approximately twice as large when using shielded crop field sensors as the reference (−1.66°C) compared to using the weather station (−0.89°C), and an order of magnitude larger (−9.26°C) when unshielded sensors were compared. Using sensors in adjacent crop fields as the baseline may better represent the thermal contrasts that organisms encounter in agricultural landscapes. Considering both types of measurements is ecologically meaningful because organisms exchange heat with their environment through radiative and convective processes. Comparing paired shielded and unshielded measurements thereby provided insight into how field margin vegetation modifies microclimates in agricultural landscapes, including its influence on radiative heating.

Our choice of HOBO loggers rather than thermocouples involved trade‐offs with measurement precision and practical deployment constraints. Thermocouples offer more accurate measurements due to their minimal thermal mass and negligible radiative heating (Maclean et al. 2021). However, our primary goal was not to measure absolute air temperature but to quantify relative temperature differences between variation in field margin vegetation structure and adjacent crop fields across multiple sites. HOBO loggers enabled a spatially distributed sensor network to capture habitat contrast and landscape‐scale variation in thermal buffering. Additionally, compact dataloggers form the basis of major microclimate databases and spatial data layers, making our measurements comparable to existing temperature mapping frameworks (Lembrechts et al. 2022, 2020). By using sensors with identical thermal properties across all sites, any radiative heating bias was consistent, preserving the validity of our comparative findings.

The substantially larger temperature differences observed when comparing field margins to adjacent crop fields, rather than weather stations, reflect the thermal conditions experienced by some organisms. Crop fields are exposed to substantial solar radiation, creating genuinely warmer microclimatic conditions that field margins buffer. Weather stations, designed to minimise solar radiation, are thermally more similar to treed field margin environments than to exposed crop fields. Thus, the thermal contrast between field margins and fields represents real environmental variation with ecological relevance, not measurement artefact. More broadly, studies relying solely on weather station comparisons may underestimate potential thermal refugia provided by vegetated features, while studies using only unshielded sensors in exposed locations capture the combined air temperature and radiative effects relevant to ectothermic organisms. The choice of measurement approach and temperature reference should therefore be aligned with the ecological process or organism being investigated. Compact temperature loggers can approximate aspects of the thermal environment experienced by some taxa, capturing fine‐scale spatial and temporal variation in temperature. For example, compact sensors exhibited thermal dynamics similar to a life‐like replica lizard (Vitt and Sartorius 1999), modified iButtons have predicted small reptile body temperatures (Vickers and Schwarzkopf 2016), and shielded HOBO temperatures strongly correlated with bumblebee surface temperatures (McFeely et al. 2026). The availability of shaded microsites allows organisms to thermoregulate and persist during extreme heat (Elmore et al. 2017; Olsen et al. 2018). Our results show that (treed) field margins provide substantial protection from solar radiation, particularly the radiative component detected by unshielded sensors, which would be underestimated if weather stations were used as the sole reference.

Our findings have practical implications for managing temperate agricultural landscapes under climate change and increasing temperature extremes. Maintaining existing treed field margins and establishing new trees in field margins can provide essential thermal refugia. Conservation strategies and land management should consider vegetation structure for both habitat and temperature regulation (Elmore et al. 2017). Vegetation structural heterogeneity is especially valuable when species' thermal requirements are poorly known, as many organisms behaviourally thermoregulate when suitable microclimates are available (Carroll et al. 2015; Elmore et al. 2017; Rakowski et al. 2019). While herbaceous field margins provide less buffering than treed margins, they likely influence below‐ and near‐ground temperatures where many organisms experience their thermal environment (Rakowski et al. 2019). Both vegetation types also affect other microclimate variables, such as humidity and light intensity, not measured in this study (Geiger et al. 1995). Beyond thermal buffering, field margins provide additional ecosystem services including habitat for beneficial insects, crop temperature moderation and carbon storage (Albrecht et al. 2020; Drever et al. 2021). Strategic establishment of treed field margins, particularly with diverse tree species, can serve as a nature‐based approach to enhancing climate resilience and creating corridors of thermally buffered habitats across agricultural landscapes (Kratschmer et al. 2024; Vanneste et al. 2020).

Forests are widely recognised as important potential climatic refugia, and their protection and expansion are commonly recommended climate change adaptation and mitigation strategies (De Frenne et al. 2019; Drever et al. 2021; IPCC 2023). Despite increasing attention to the biodiversity benefits of field margins and small patches (Priyadarshana et al. 2024; Riva and Fahrig 2022), their capacity to buffer temperatures in temperate agricultural settings has received less focus. Given projected increases in summer daily maximum temperature of 4.6°C by 2080 in this region (https://climateatlas.ca/map/canada), the magnitude of cooling provided by treed hedgerows could be ecologically meaningful. Alongside other potential co‐benefits, including crop temperature moderation and carbon sequestration, maintaining and increasing field margin tree cover may benefit biodiversity and agriculture under climate change. Overall, treed field margins enhance microclimatic buffering and have the potential to support climate adaptation in temperate agricultural landscapes.

## Author Contributions

**Sarah Endicott:** data curation (equal), formal analysis (equal), methodology (equal), visualization (equal), writing – original draft (equal), writing – review and editing (equal). **Adam Blasl:** conceptualization (supporting), formal analysis (equal), investigation (equal), methodology (equal), writing – original draft (equal), writing – review and editing (supporting). **Marie‐Hélène Brice:** formal analysis (equal), methodology (equal), visualization (equal), writing – review and editing (equal). **Ilona Naujokaitis‐Lewis:** conceptualization (lead), funding acquisition (lead), investigation (equal), methodology (equal), project administration (lead), resources (lead), supervision (lead), writing – original draft (equal), writing – review and editing (lead).

## Funding

The authors have nothing to report.

## Conflicts of Interest

The authors declare no conflicts of interest.

## Supporting information


**Table S1.** Summary of temperatures recorded by unshielded and shielded sensors located in field margins and crop fields, as well as inverse distance weighted mean temperatures for each field site based on weather stations within 30 km (*n* = 7). Mean, median, standard deviation (SD) and interquartile range (IQR) were calculated across all sites and days in the sample. Daily average absolute deviation (AAD) includes data from 4 weather stations because hourly data was not available for all stations.
**Table S2.** Results of paired Wilcoxon signed rank tests for daily temperature metrics with shielded and unshielded sensors as well as tall field margins (≥ 7.5 m) and short field margins (< 7.5 m) for all combinations of field, hedgerow and macroclimate (Macro).
**Table S3.** Temperature offset model results for shielded sensors with field margin height included as a binary variable.
**Table S4.** Temperature model results for unshielded sensors with field margin height included as a binary variable.
**Table S5.** Temperature offset model results for shielded sensors with field margin height included as a continuous variable.
**Table S6.** Temperature offset model results for unshielded sensors with field margin height included as a continuous variable.
**Figure S1.** Histograms showing the distribution of the covariates used in the model.
**Figure S2.** (a) Median $T_{\text{offset}}$ ($T_{\text{hedgerow}}-T_{\text{field}}$) based on paired Wilcoxon signed rank tests for daily temperature metrics with shielded and unshielded sensors. (b) Compares sensors located in tall and short field margins.
**Figure S3.** Median offsets between the microclimate and the macroclimate based on paired Wilcoxon signed rank tests for daily temperature metrics with shielded and unshielded sensors. (a) Offsets for sensors located in fields and in field margins (b) Offsets for sensors located in tall field margins and short field margins.
**Figure S4.** Plots of the autocorrelation function for the normalised residuals of models fit with (1) no correlation structure, (2) an auto‐regressive model of order 1 (corAR1) auto‐correlation structure and (3) an autocorrelation‐moving average correlation structure of order *p* = *q* = 1 (corARMA).

## Data Availability

All data and code required to reproduce this analysis is available at https://osf.io/4d9km/?view_only=432120fe42bf4d07b617357825f102d3.
