# Supplementary material for: Field Margin Vegetation Structure Buffers Microclimate Extremes in Temperate Agricultural Landscapes
Source: Ecol Evol. 2026 Aug 18;16(8):e73899. doi: 10.1002/ece3.73899 (PMC13482773; doi:10.1002/ece3.73899)
Supplement: Supplementary file 1 [file ECE3-16-e73899-s001.docx]

## Supplementary Material

## Tables

**Table S1**: Summary of temperatures recorded by unshielded and shielded sensors located in hedgerows and field centres, as well as inverse distance weighted mean temperatures for each field site based on weather stations within 30 km (n = 7). Mean, median, standard deviation (SD) and interquartile range (IQR) were calculated across all sites and days in the sample. Daily average absolute deviation (AAD) includes data from 4 weather stations because hourly data was not available for all stations.

| Metric | Position | Mean | Median | SD | IQR |
| --- | --- | --- | --- | --- | --- |
| Daily min | Margin - unshielded | 14.64 | 15.06 | 4.41 | 6.70 |
| Daily min | Field - unshielded | 13.66 | 13.92 | 4.62 | 7.28 |
| Daily min | Margin - shielded | 14.79 | 15.18 | 4.34 | 6.61 |
| Daily min | Field - shielded | 14.24 | 14.52 | 4.50 | 7.12 |
| Daily min | Weather station | 15.44 | 15.83 | 3.97 | 5.76 |
| Daily mean | Margin - unshielded | 22.70 | 22.93 | 3.67 | 4.29 |
| Daily mean | Field - unshielded | 24.24 | 24.30 | 3.70 | 4.16 |
| Daily mean | Margin - shielded | 21.26 | 21.47 | 3.31 | 3.93 |
| Daily mean | Field - shielded | 21.48 | 21.68 | 3.38 | 4.06 |
| Daily mean | Weather station | 21.65 | 21.83 | 3.49 | 4.32 |
| Daily max | Margin - unshielded | 32.58 | 31.32 | 6.59 | 10.10 |
| Daily max | Field - unshielded | 38.03 | 38.93 | 5.09 | 6.63 |
| Daily max | Margin - shielded | 27.66 | 27.58 | 3.77 | 4.46 |
| Daily max | Field - shielded | 28.73 | 28.87 | 3.65 | 4.33 |
| Daily max | Weather station | 27.83 | 28.16 | 3.77 | 4.66 |
| Daily AAD | Margin - unshielded | 6.90 | 6.19 | 3.92 | 4.80 |
| Daily AAD | Field - unshielded | 9.35 | 9.04 | 4.26 | 6.77 |
| Daily AAD | Margin - shielded | 5.51 | 5.18 | 2.66 | 3.82 |
| Daily AAD | Field - shielded | 6.20 | 6.04 | 2.80 | 4.29 |
| Daily AAD | Weather station | 3.62 | 3.36 | 1.49 | 2.36 |

**Table S2**: Results of paired Wilcoxon signed rank tests for daily temperature metrics with shielded and unshielded sensors as well as tall hedgerows and short hedgerows for all combinations of field, hedgerow and macroclimate (Macro).

| Measure | Shielded? | Margin height | Sensor 1 | Sensor 2 | Estimated median difference | Lower CI | Upper CI | N | Statistic | p-value | Adjusted p-value |
| --- | --- | --- | --- | --- | --- | --- | --- | --- | --- | --- | --- |
| Daily min | No | All | Margin | Field | 0.956 | 0.909 | 1.006 | 2293 | 2312226.0 | < 0.0001 | < 0.0001 |
| Daily min | No | All | Margin | Weather station | -0.707 | -0.765 | -0.650 | 2293 | 545751.0 | < 0.0001 | < 0.0001 |
| Daily min | No | All | Field | Weather station | -1.740 | -1.797 | -1.683 | 2293 | 50632.0 | < 0.0001 | < 0.0001 |
| Daily min | Yes | All | Margin | Field | 0.493 | 0.450 | 0.536 | 2081 | 1695911.5 | < 0.0001 | < 0.0001 |
| Daily min | Yes | All | Margin | Weather station | -0.570 | -0.621 | -0.519 | 2081 | 486244.0 | < 0.0001 | < 0.0001 |
| Daily min | Yes | All | Field | Weather station | -1.139 | -1.195 | -1.082 | 2081 | 181393.0 | < 0.0001 | < 0.0001 |
| Daily mean | No | All | Margin | Field | -1.548 | -1.614 | -1.480 | 2293 | 258726.0 | < 0.0001 | < 0.0001 |
| Daily mean | No | All | Margin | Weather station | 0.895 | 0.810 | 0.980 | 2293 | 2038919.0 | < 0.0001 | < 0.0001 |
| Daily mean | No | All | Field | Weather station | 2.608 | 2.541 | 2.674 | 2293 | 2605325.0 | < 0.0001 | < 0.0001 |
| Daily mean | Yes | All | Margin | Field | -0.187 | -0.205 | -0.170 | 2081 | 504091.0 | < 0.0001 | < 0.0001 |
| Daily mean | Yes | All | Margin | Weather station | -0.416 | -0.451 | -0.381 | 2081 | 522472.0 | < 0.0001 | < 0.0001 |
| Daily mean | Yes | All | Field | Weather station | -0.194 | -0.228 | -0.159 | 2081 | 797597.0 | < 0.0001 | < 0.0001 |
| Daily max | No | All | Margin | Field | -5.495 | -5.744 | -5.253 | 2293 | 266066.5 | < 0.0001 | < 0.0001 |
| Daily max | No | All | Margin | Weather station | 4.638 | 4.372 | 4.890 | 2293 | 2399266.0 | < 0.0001 | < 0.0001 |
| Daily max | No | All | Field | Weather station | 10.209 | 10.077 | 10.339 | 2293 | 2630071.0 | < 0.0001 | < 0.0001 |
| Daily max | Yes | All | Margin | Field | -0.987 | -1.051 | -0.922 | 2081 | 276877.0 | < 0.0001 | < 0.0001 |
| Daily max | Yes | All | Margin | Weather station | -0.236 | -0.296 | -0.175 | 2081 | 881164.0 | < 0.0001 | < 0.0001 |
| Daily max | Yes | All | Field | Weather station | 0.790 | 0.743 | 0.839 | 2081 | 1907849.0 | < 0.0001 | < 0.0001 |
| Daily min | No | < 7.5 m | Margin | Weather station | -1.818 | -1.913 | -1.726 | 875 | 5123.0 | < 0.0001 | < 0.0001 |
| Daily min | No | < 7.5 m | Margin | Field | -0.024 | -0.052 | 0.003 | 875 | 168841.5 | 0.105 | 0.105 |
| Daily min | No | ≥ 7.5 m | Margin | Weather station | -0.132 | -0.179 | -0.084 | 1418 | 419414.0 | < 0.0001 | < 0.0001 |
| Daily min | No | ≥ 7.5 m | Margin | Field | 1.579 | 1.532 | 1.629 | 1418 | 1003385.0 | < 0.0001 | < 0.0001 |
| Daily min | Yes | < 7.5 m | Margin | Weather station | -1.232 | -1.318 | -1.146 | 821 | 18415.0 | < 0.0001 | < 0.0001 |
| Daily min | Yes | < 7.5 m | Margin | Field | -0.043 | -0.086 | -0.021 | 821 | 137966.5 | 0.002 | 0.002 |
| Daily min | Yes | ≥ 7.5 m | Margin | Weather station | -0.192 | -0.243 | -0.142 | 1260 | 301231.0 | < 0.0001 | < 0.0001 |
| Daily min | Yes | ≥ 7.5 m | Margin | Field | 0.901 | 0.858 | 0.944 | 1260 | 741719.0 | < 0.0001 | < 0.0001 |
| Daily mean | No | < 7.5 m | Margin | Weather station | 2.721 | 2.617 | 2.824 | 875 | 381347.0 | < 0.0001 | < 0.0001 |
| Daily mean | No | < 7.5 m | Margin | Field | 0.008 | -0.031 | 0.048 | 875 | 194519.5 | 0.699 | 0.699 |
| Daily mean | No | ≥ 7.5 m | Margin | Weather station | -0.017 | -0.062 | 0.028 | 1418 | 491450.0 | 0.453 | 0.453 |
| Daily mean | No | ≥ 7.5 m | Margin | Field | -2.580 | -2.644 | -2.516 | 1418 | 1128.0 | < 0.0001 | < 0.0001 |
| Daily mean | Yes | < 7.5 m | Margin | Weather station | -0.203 | -0.259 | -0.146 | 821 | 123105.0 | < 0.0001 | < 0.0001 |
| Daily mean | Yes | < 7.5 m | Margin | Field | -0.013 | -0.029 | 0.002 | 821 | 157047.0 | 0.086 | 0.086 |
| Daily mean | Yes | ≥ 7.5 m | Margin | Weather station | -0.553 | -0.597 | -0.510 | 1260 | 132813.0 | < 0.0001 | < 0.0001 |
| Daily mean | Yes | ≥ 7.5 m | Margin | Field | -0.333 | -0.358 | -0.309 | 1260 | 101965.0 | < 0.0001 | < 0.0001 |
| Daily max | No | < 7.5 m | Margin | Weather station | 10.692 | 10.477 | 10.909 | 875 | 383250.0 | < 0.0001 | < 0.0001 |
| Daily max | No | < 7.5 m | Margin | Field | 0.520 | 0.332 | 0.715 | 875 | 233316.0 | < 0.0001 | < 0.0001 |
| Daily max | No | ≥ 7.5 m | Margin | Weather station | 0.877 | 0.779 | 0.979 | 1418 | 776244.0 | < 0.0001 | < 0.0001 |
| Daily max | No | ≥ 7.5 m | Margin | Field | -9.265 | -9.424 | -9.106 | 1418 | 2.0 | < 0.0001 | < 0.0001 |
| Daily max | Yes | < 7.5 m | Margin | Weather station | 0.797 | 0.719 | 0.874 | 821 | 289611.0 | < 0.0001 | < 0.0001 |
| Daily max | Yes | < 7.5 m | Margin | Field | 0.000 | -0.043 | 0.043 | 821 | 158181.0 | 0.9 | 0.9 |
| Daily max | Yes | ≥ 7.5 m | Margin | Weather station | -0.885 | -0.938 | -0.832 | 1260 | 100890.0 | < 0.0001 | < 0.0001 |
| Daily max | Yes | ≥ 7.5 m | Margin | Field | -1.652 | -1.718 | -1.587 | 1260 | 10224.0 | < 0.0001 | < 0.0001 |

**Table S3:** Temperature offset model results for shielded sensors with hedgerow height included as a binary variable.

|  | Temperature offset | | |
| --- | --- | --- | --- |
|  | Daily min | Daily mean | Daily max |
|  | | | |
| Margin height (Tall) | 0.833^***^ | -0.316^***^ | -1.373^***^ |
|  | (0.369, 1.296) | (-0.506, -0.126) | (-2.206, -0.540) |
| Margin width | 0.207 | 0.058 | -0.375 |
|  | (-0.201, 0.616) | (-0.109, 0.225) | (-1.110, 0.360) |
| % natural | -0.066 | -0.015 | -0.253 |
|  | (-0.590, 0.457) | (-0.229, 0.199) | (-1.194, 0.689) |
| Macroclimate | -0.225^***^ | -0.051^***^ | -0.087^**^ |
|  | (-0.262, -0.188) | (-0.076, -0.027) | (-0.156, -0.017) |
| Margin height (Tall):Margin width | -0.032 | -0.064 | 0.270 |
|  | (-0.504, 0.439) | (-0.257, 0.128) | (-0.579, 1.118) |
| % natural:Margin height (Tall) | -0.169 | -0.135 | 0.082 |
|  | (-0.769, 0.432) | (-0.380, 0.110) | (-0.998, 1.162) |
| % natural:Margin width | 0.073 | 0.012 | -0.179 |
|  | (-0.123, 0.269) | (-0.067, 0.092) | (-0.532, 0.175) |
| Constant | -0.262 | -0.015 | -0.168 |
|  | (-0.657, 0.133) | (-0.176, 0.146) | (-0.875, 0.540) |
|  | | | |
| Random Effects: |  |  |  |
| Residual variance | 0.5 | 0.14 | 1.08 |
| Intercept variance | 0 | 0 | 0.4 |
| ICC | 0 | 0 | 0.27 |
| N sites | 27 | 27 | 27 |
| Model statistics: |  |  |  |
| Marginal R-squared | 0.343 | 0.251 | 0.384 |
| Observations | 2,081 | 2,081 | 2,081 |
| Log Likelihood | -1,552.347 | -256.242 | -2,595.748 |
| Akaike Inf. Crit. | 3,128.694 | 536.484 | 5,215.497 |
|  | | | |
| *Note:* | ^*^p<0.1; ^**^p<0.05; ^***^p<0.01 | | |

**Table S4:** Temperature model results for unshielded sensors with hedgerow height included as a binary variable.

|  | Temperature offset | | |
| --- | --- | --- | --- |
|  | Daily min | Daily mean | Daily max |
|  | | | |
| Margin height (Tall) | 1.698^***^ | -2.669^***^ | -9.798^***^ |
|  | (1.330, 2.066) | (-3.170, -2.167) | (-11.516, -8.080) |
| Margin width | -0.028 | -0.127 | -0.677 |
|  | (-0.368, 0.311) | (-0.586, 0.332) | (-2.258, 0.904) |
| % natural | -0.215 | -0.052 | -0.390 |
|  | (-0.638, 0.209) | (-0.627, 0.523) | (-2.366, 1.585) |
| Macroclimate | -0.389^***^ | -0.015 | -0.458^***^ |
|  | (-0.433, -0.345) | (-0.082, 0.053) | (-0.633, -0.282) |
| Margin height (Tall):Margin width | 0.170 | 0.105 | 0.740 |
|  | (-0.220, 0.561) | (-0.423, 0.633) | (-1.079, 2.560) |
| % natural:Margin height (Tall) | 0.091 | 0.045 | 0.589 |
|  | (-0.393, 0.575) | (-0.611, 0.701) | (-1.669, 2.847) |
| % natural:Margin width | -0.075 | 0.104 | 0.170 |
|  | (-0.236, 0.087) | (-0.113, 0.321) | (-0.582, 0.923) |
| Constant | -0.491^***^ | 0.084 | 0.746 |
|  | (-0.805, -0.176) | (-0.342, 0.511) | (-0.719, 2.211) |
|  | | | |
| Random Effects: |  |  |  |
| Residual variance | 0.42 | 1.1 | 7.66 |
| Intercept variance | 0.1 | 0 | 1.57 |
| ICC | 0.19 | 0 | 0.17 |
| N sites | 30 | 30 | 30 |
| Model statistics: |  |  |  |
| Marginal R-squared | 0.564 | 0.615 | 0.726 |
| Observations | 2,293 | 2,293 | 2,293 |
| Log Likelihood | -1,981.438 | -2,673.447 | -5,033.326 |
| Akaike Inf. Crit. | 3,986.877 | 5,370.895 | 10,090.650 |
| Bayesian Inf. Crit. | 4,055.686 | 5,439.704 | 10,159.460 |
|  | | | |
| *Note:* | ^*^p<0.1; ^**^p<0.05; ^***^p<0.01 | | |

**Table S5:** Temperature offset model results for shielded sensors with hedgerow height included as a continuous variable.

|  | Temperature offset | | |
| --- | --- | --- | --- |
|  | Daily min | Daily mean | Daily max |
| Margin height | 0.430^***^ | -0.101^**^ | -0.653^***^ |
|  | (0.248, 0.612) | (-0.180, -0.022) | (-0.955, -0.352) |
| Margin height squared | -0.223^*^ | 0.082 | 0.522^**^ |
|  | (-0.454, 0.009) | (-0.018, 0.183) | (0.139, 0.905) |
| Margin width | 0.089 | 0.016 | -0.092 |
|  | (-0.114, 0.292) | (-0.072, 0.103) | (-0.428, 0.244) |
| % natural | -0.177 | -0.127^**^ | -0.187 |
|  | (-0.382, 0.028) | (-0.216, -0.039) | (-0.526, 0.152) |
| Macroclimate | -0.225^***^ | -0.051^***^ | -0.087^**^ |
|  | (-0.262, -0.188) | (-0.076, -0.026) | (-0.156, -0.017) |
| Margin height:Margin width | 0.114 | -0.026 | -0.060 |
|  | (-0.091, 0.319) | (-0.114, 0.062) | (-0.399, 0.279) |
| % natural:Margin height | -0.106 | 0.0004 | 0.109 |
|  | (-0.340, 0.128) | (-0.101, 0.101) | (-0.278, 0.496) |
| % natural:Margin width | 0.118 | -0.022 | -0.235 |
|  | (-0.056, 0.292) | (-0.097, 0.053) | (-0.523, 0.052) |
| Constant | 0.409^***^ | -0.303^***^ | -1.467^***^ |
|  | (0.140, 0.677) | (-0.418, -0.188) | (-1.910, -1.024) |
|  | | | |
| Random Effects: |  |  |  |
| Residual variance | 0.5 | 0.14 | 1.09 |
| Intercept variance | 0 | 0 | 0.29 |
| ICC | 0 | 0 | 0.21 |
| N sites | 27 | 27 | 27 |
| Model statistics: |  |  |  |
| Marginal R-squared | 0.355 | 0.235 | 0.425 |
| Observations | 2,081 | 2,081 | 2,081 |
| Log Likelihood | -1,555.488 | -262.172 | -2,596.686 |
| Akaike Inf. Crit. | 3,136.977 | 550.344 | 5,219.371 |
|  | | | |
| *Note:* | ^*^p<0.1; ^**^p<0.05; ^***^p<0.01 | | |

**Table S6:** Temperature offset model results for unshielded sensors with hedgerow height included as a continuous variable.

|  | Temperature offset | | |
| --- | --- | --- | --- |
|  | Daily min | Daily mean | Daily max |
|  | | | |
| Margin height | 0.720^***^ | -1.254^***^ | -4.701^***^ |
|  | (0.515, 0.925) | (-1.556, -0.952) | (-5.757, -3.645) |
| Margin height squared | -0.421^***^ | 0.312 | 1.774^**^ |
|  | (-0.676, -0.166) | (-0.064, 0.689) | (0.459, 3.090) |
| Margin width | 0.008 | 0.062 | 0.335 |
|  | (-0.223, 0.239) | (-0.279, 0.402) | (-0.854, 1.525) |
| % natural | -0.147 | 0.019 | 0.226 |
|  | (-0.377, 0.082) | (-0.320, 0.357) | (-0.958, 1.411) |
| Macroclimate | -0.389^***^ | -0.013 | -0.456^***^ |
|  | (-0.433, -0.345) | (-0.080, 0.054) | (-0.631, -0.281) |
| Margin height:Margin width | 0.176 | -0.054 | -0.291 |
|  | (-0.057, 0.409) | (-0.397, 0.288) | (-1.489, 0.908) |
| % natural:Margin height | -0.034 | 0.060 | 0.325 |
|  | (-0.302, 0.233) | (-0.333, 0.454) | (-1.052, 1.701) |
| % natural:Margin width | 0.056 | -0.087 | -0.449 |
|  | (-0.143, 0.256) | (-0.381, 0.207) | (-1.478, 0.579) |
| Constant | 0.888^***^ | -1.746^***^ | -6.493^***^ |
|  | (0.605, 1.171) | (-2.160, -1.332) | (-7.943, -5.042) |
|  | | | |
| Random Effects: |  |  |  |
| Residual variance | 0.42 | 1.19 | 7.67 |
| Intercept variance | 0.2 | 0.21 | 5.01 |
| ICC | 0.33 | 0.15 | 0.4 |
| N sites | 30 | 30 | 30 |
| Model statistics: |  |  |  |
| Marginal R-squared | 0.49 | 0.522 | 0.636 |
| Observations | 2,293 | 2,293 | 2,293 |
| Log Likelihood | -1,991.952 | -2,687.741 | -5,044.579 |
| Akaike Inf. Crit. | 4,009.905 | 5,401.483 | 10,115.160 |
| Bayesian Inf. Crit. | 4,084.443 | 5,476.021 | 10,189.700 |
|  | | | |
| *Note:* | ^*^p<0.1; ^**^p<0.05; ^***^p<0.01 | | |

## Figures

**Figure S1:** Histograms showing the distribution of the covariates used in the model.

**Figure S2:** a) Median $T_{offset}$ *(*$T_{hedgerow}-T_{field}$*)* based on paired Wilcoxon signed rank tests for daily temperature metrics with shielded and unshielded sensors. b) Compares sensors located in tall hedgerows and short hedgerows.

**Figure S3:** Median offsets between the microclimate and the macroclimate based on paired Wilcoxon signed rank tests for daily temperature metrics with shielded and unshielded sensors. a) Offsets for sensors located in fields and in field margins b) Offsets for sensors located in tall field margins and short field margins.

**Figure S4:** Plots of the autocorrelation function for the normalized residuals of models fit with (1) no correlation structure, (2) an auto-regressive model of order 1 (corAR1) auto-correlation structure, and (3) an autocorrelation-moving average correlation structure of order p = q = 1 (corARMA).
